# Genetic counseling and carrier screening in candidates for gamete donation at a Portuguese center

**DOI:** 10.5935/1518-0557.20220012

**Published:** 2023

**Authors:** Célia Azevedo Soares, Natália Tkachenko, Emídio Vale Fernandes, Márcia Barreiro, Maria Abreu, Cláudia Falcão Reis, Gabriela Soares, Ana Maria Fortuna, Ana Rita Soares

**Affiliations:** 1 Serviço de Genética Médica, Centro de Genética Médica Jacinto Magalhães, Centro Hospitalar Universitário do Porto, Porto, Portugal; 2 Unit for Multidisciplinary Research in Biomedicine, Instituto de Ciências Biomédicas Abel Salazar/Universidade do Porto, Porto, Portugal; 3 Centro de Procriação Medicamente Assistida / Banco Público de Gâmetas, Serviço de Ginecologia - Departamento da Mulher e da Medicina Reprodutiva, Centro Materno-Infantil do Norte, Centro Hospitalar Universitário do Porto, Porto, Portugal; 4 Life and Health Sciences Research Institute (ICVS), University of Minho, Campus de Gualtar, Braga 4710-057, Portugal; ICVS/3B’s, PT Government Associate Laboratory, Braga, Guimarães, Portugal; Clinical Academic Center, Braga, Portugal; 5 Consulta de Genética Médica, Hospital São Pedro em Vila Real, Centro Hospitalar de Trás-os-Montes e Alto Douro, Vila Real, Portugal

**Keywords:** carrier screening, gamete donation, genetic counseling

## Abstract

**Objective:**

Genetic counseling and carrier screening are part of the gamete donation process by healthy individuals. We aim to review the findings of genetic counseling and carrier screening of a cohort of candidates at our public gametes bank.

**Methods:**

Thirty-four male and 64 female candidates had genetic counseling with a medical geneticist before donation. Of these, one female candidate voluntarily dropped-out. Thirty-four males and 63 females performed karyotype and screening for the more common pathogenic variants for *CFTR*-related cystic fibrosis and spinal muscular atrophy (*SMN1*) in the Portuguese population. In addition, all females also performed Fragile X expansion screening (*FMR1*). Thirty candidates with known or assumed African ancestry performed hemoglobinopathies screening.

**Results:**

Six candidates were definitely or temporarily withheld from the donation process given their family or personal history that required further investigation. Of 97 candidates tested, 16.5% presented anomalous laboratory results (16/97): ten candidates were carriers for an autosomal recessive disorder - cystic fibrosis (5/97), sickle cell anemia (3/30), and spinal muscular atrophy (2/97). One female was an *FMR1* pre-mutation carrier (1/63). One female candidate presented with triple X mosaicism: 47,XXX[2]/46,XX[50]. Two candidates presented with chromosomal instability of unknown origin. In one candidate, a mosaic for the Philadelphia chromosome was detected, revealing the diagnosis of chronic myeloid leukemia.

**Conclusions:**

From a cohort of 97 candidates, 21.7% had a family/personal history or an anomalous laboratory result that required additional genetic counseling, stressing the importance of performing pre-donation genetic counseling in this population.

## INTRODUCTION

Genetic counseling and carrier screening are initial steps in the process of anonymous gamete donation by healthy individuals. These steps include two components: exploring the medical and family history of the candidate donor and perform genetic carrier screening, to decrease the risk of severe genetic disorders in donor-conceived offspring (Practice Committee of the American Society for Reproductive Medicine & the Practice Committee of the Society for Assisted Reproductive Technology, 2021).

Most professional medical societies involved in fertility and reproduction agree on the relevance of pre-test genetic counseling, but they differ in the strategy for genetic carrier screening. Previous recommendations by the American Society for Reproductive Medicine (ASRM) and the Society for Assisted Reproductive Technology (SART) (Practice Committee of the American Society for Reproductive Medicine & the Practice Committee of the Society for Assisted Reproductive Technology, 2013) recommended carrier screening for *CFTR-*related cystic fibrosis and spinal muscular atrophy (SMA -gene *SMN1)* common variants in all donors. Routine karyotyping of donors was considered optional. Further carrier screening should be directed to donor´s ancestry, such as Tay-Sachs in Ashkenazy Jews or hemoglobinopathies in donors with African or South Mediterranean ancestry. A 2021 update of these guidelines (Practice Committee of the American Society for Reproductive Medicine & the Practice Committee of the Society for Assisted Reproductive Technology, 2021) expanded hemoglobinopathies to all candidates. This update also states that screening for *FMR1* CGG expansion, causal of Fragile X syndrome, should be considered in all oocyte donors, regardless of the family history, and should be performed in female candidates with a positive family history for Fragile X syndrome. The European Society of Human Reproduction and Embryology (ESHRE) has a similar recommendation (Dondorp *et al*., 2014) for the disorders screened supported by the European Commission 2006 directive recommending screening for the most prevalent autosomal recessive disorders (European Parliament, 2006). Nevertheless, this society stresses the risks and benefits of screening for Fragile-X syndrome *FMR1* CGG expansion and routine karyotyping.

In this manuscript, we present the findings of genetic counseling and basic carrier screening of a cohort of candidates for gamete donation. We report that often further studies or counseling were necessary to address issues related with findings from genetic screening, but also from personal and familial history. Future standard operation procedures in gamete donation genetic counseling and carrier screening should consider how frequent and complex these findings are, and prepare the health professionals to deal with the burden of these findings to the patient and healthcare system.

## MATERIALS AND METHODS

### Study design

This study was reviewed and approved by the Centro Hospitalar Universitário do Porto (CHUPorto) Ethical Committee with approval 2021.021 (016-DEFI/016-CE), in accordance with the 1975 Helsinki declaration, reviewed in 2013. Gamete donors were referred to our Medical Genetics appointment after an initial evaluation at the Assisted Medical Reproduction Unit at CHUPorto. Standard procedures in the donation process not related with genetics were performed by that unit. The data presented in this study is relative to the appointments performed between January 2019 and March 2020. These appointments were suspended from March 2020 to March 2021 due to the SARSCOVID-19 pandemic. Candidates´ information was recorded in a specific physical record, only accessible by medical geneticists. Consent form and results were also kept in a separate file. Donation application was not mentioned in electronic health records. Anonymized data was collected and processed on Stata^®^ version 13 (College Station, TX, USA).

### Genetic counseling

Genetic counseling was performed and carrier screening was offered. Further studies could be offered directed to candidates’ personal and family history. Candidates who consented for testing signed a written consent form. In the consent form, candidates could opt not to know the result of the carrier screening. Candidates with no findings received results and reinforcement of counseling by a phone call, and a written report was sent to their preferential address. Candidates with alterations had a new appointment to disclose the results and initiate directed genetic counseling. If necessary, a letter was emitted inviting first-degree family members for genetic counseling, and given to the candidate.

### Genetic screening

For the carrier screening, high-resolution G-banding karyotype was performed. For *CFTR-*related cystic fibrosis screening, CF-EU2v1 and Iberian panels, which screen for the most common pan-European and Iberian pathogenic variants, were used in all candidates (Elucigene^®^). Pathogenic copy number variants in *SMN1* exon 7-8 were screened with SALSA^®^ MLPA^®^ Probemix P021-B1 SMA (MRC Holland) and the variant *SMN1* c.770_780dup by polymerase chain reaction (PCR). For females, Amplidex^®^ kit was used to characterize CGG repeats in *FMR1* 5´UTR (Jorge *et al*., 2013). Hemoglobinopathies were screened by electrophoresis and complete blood count.

## RESULTS

### Candidates for donation

**Figure 1:**
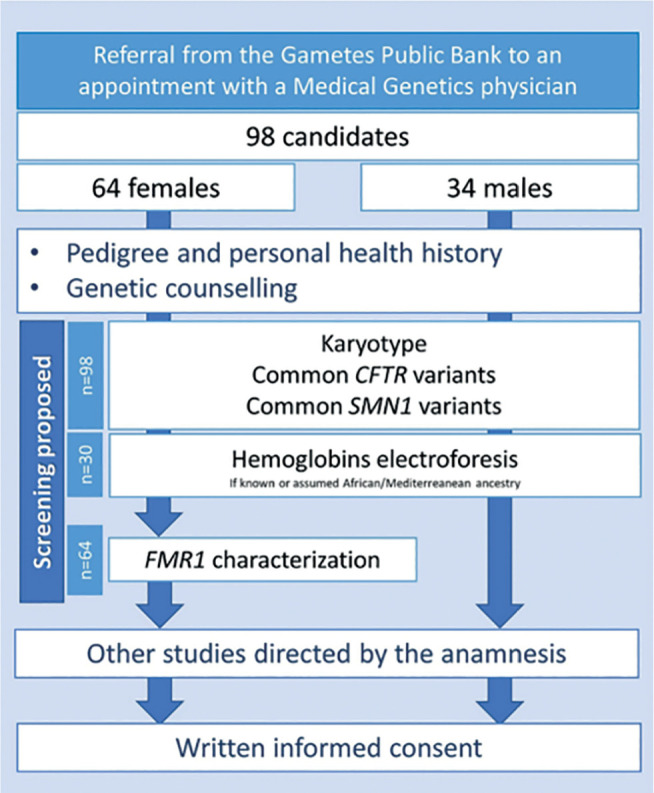
Standard operation procedures for the genetic counselling and carrier screening at our unit from January 2019 to March 2020.

**Figure 2:**
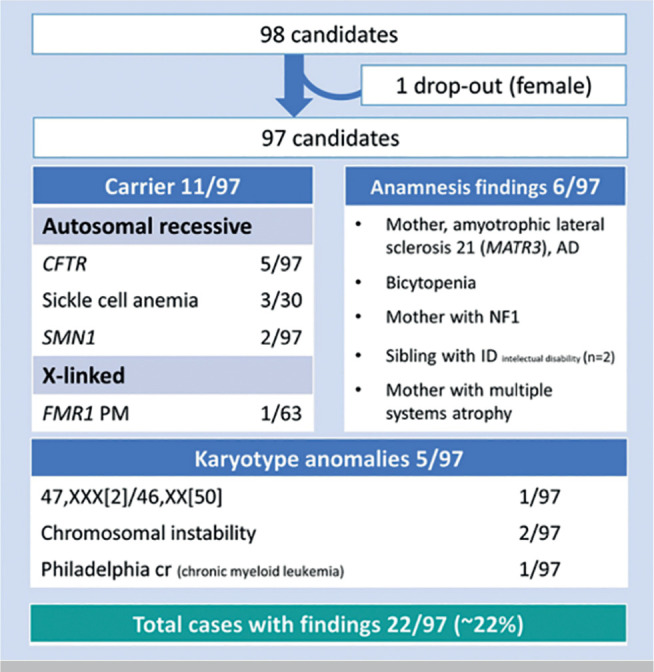
Findings in the genetic counselling and carrier screening in our cohort.

Thirty-four male and 64 female candidates initiated the genetic counseling process. Candidates’ ancestry was Portuguese, Brazilian, and Cape Verdian. Candidates’ median age was 28 years old, with the youngest candidate being 18 and the eldest 39. All candidates accepted genetic screening and signed a consent form for testing. In selected cases, further information was requested on the health of self or family members (Figure 1). One female candidate dropped out after the first appointment due to ongoing pregnancy (Figure 2). Thirty-four males and 63 females performed karyotype and screening for the more common pathogenic variants for SMA and *CFTR*-related cystic fibrosis. In addition, all females also performed *FMR1* study. Thirty candidates with African and/or Mediterranean ancestry performed hemoglobinopathies screening (Figure 1).

### Personal and familial history elicited further investigations in some cases

From an initial cohort of 98 candidates, six presented a relevant finding in their personal or familial history (Figure 2). One female candidate had a personal history of bicytopenia, being referred for Hematology, and missed this appointment. This candidate did not advance in the donation process due to loss to follow-up. Five candidates had a relevant family history. Two candidates had siblings affected by intellectual disability (ID), with their donation being upheld until further etiological studies clarified the risk of recurrence of ID in the candidates’ offspring. One candidate’s mother had the diagnosis of neurofibromatosis type 1 (NF1), an autosomal dominant multisystemic disorder, whose features are usually present by late adolescence-early adulthood. Given that the candidate did not present clinical features of NF1 in adulthood, the familial diagnosis was excluded in the candidate, and she proceeded with the donation process. One candidate’s mother had *MATR3-*related amyotrophic lateral sclerosis 21, an adult-onset autosomal dominant disorder, and another candidate´s mother had a neurological disorder under investigation. These two candidates voluntarily withdrew from the donation process and continued in our clinic for directed genetic counseling.

### Genetic screening was abnormal in 16.5% of candidates

On the carrier screening for autosomal recessive and X-linked recessive disorders (Figure 2), 11/97 candidates had a heterozygous pathogenic variant, representing 11.3% of candidates tested (*CFTR*-related cystic fibrosis n=5/97, hemoglobinopathy-sickle cell anemia n=3/30, spinal muscular atrophy n=2/97, *FMR1* premutation n=1/63).

Karyotype was abnormal in five candidates from a group of 97. One male candidate presented a recurrent fragile site in chromosome 16 with no increase of chromosomal instability after diepoxybutane (DEB) induction. A female candidate had a karyotype suggestive of chromosomal instability, a finding not replicated in the DEB induction. One female candidate with low levels of anti-mullerian hormone presented low mosaicism for X chromosome aneuploidy: 47,XXX[2]/46,XX[50]. One male candidate in a subset of cells from peripheral blood had a t(9;22)(q34;q11), known as the Philadelphia chromosome, usually present in some forms of blood cancer. This candidate was referred to Hematology where he was diagnosed with chronic myeloid leukemia (CML) and started treatment before developing clinical symptoms.

Overall, in the genetic screening, 16/97 candidates had an alteration, representing 16.5% of the candidates tested.

### Extended counseling in family members

For autosomal and X-linked recessive disorders, further counseling of first-degree family members was recommended. Of 11 candidates with this recommendation, four families started cascade genetic counseling for a known familial variant. The context of initial testing in the family member was not disclosed, but it was often already known by the family members by the initiative of the index case. Some candidates did not plan to start familial screening in our clinic, due to the fact that their family members lived abroad (n=4/11).

For a candidate with a mother with *MATR3-*related amyotrophic lateral sclerosis 21, further follow-up was initiated, and it was proposed to start pre-symptomatic counseling protocol.

For a candidate with a mother with NF1, it was recommended extending the NF1-specific counseling to all affected family members.

## DISCUSSION

In the present study, we verified that the frequency of findings in genetic counseling and carrier screening of healthy candidates for gamete donation was relatively high with 21.7% of candidates having a finding that elicited further counseling or testing. Our values are higher than previous studies that only considered the findings of carrier screening. These results support the need for specialized pre-test and post-test counseling to deal with these findings and the psychological burden to the candidates (Amor *et al*., 2018).

Genetic counseling in the gamete donation process includes the process of explaining genetic inheritance and the contribution of genetic factors to disease and exploring indicators of a high penetrance genetic disorder in a donor´s personal or familial history (Castilla *et al*., 2020). The health professional responsible for this counseling varies in different countries, with physicians, nurses, or genetic counselors delivering this information, with or without specific training in this field (Dondorp *et al*., 2014; Urbina *et al*., 2017). Under the Portuguese Law 12/2005 (Assembleia da República, 2005; Ministério da Saúde, 2014), genetic counseling and carrier screening are activities only performed by a physician with training in Medical Genetics. In our cohort, having the counseling been performed by medical geneticists, there was a significant identification of red flags in the personal and family history with prompt orientation to standard care. In one patient at risk of being affected by NF1, the physical examination by a trained professional on the first appointment accelerated the donation process and guaranteed a prompt specialized genetic counseling. Nevertheless, the access to genetic counseling by trained professionals is a challenge worldwide, with many countries not recognizing the genetic counselor profession. In the coming years, the health care professionals involved in the gamete donation process should reflect about the competence level that should be required to the professionals performing the genetic counseling and carrier screening in the gamete donation candidates (Urbina *et al*., 2017), with the active participation of donors and recipients (Amor *et al*., 2018).

For many years the standard procedure for carrier screening was directed to a short pan-ethnic list of genes and additional studies directed to ethnic background. Genes to screen were chosen by the criteria of high carrier frequency in a population, well-defined phenotype, high penetrance, and severe impact on the health of donor-conceived offspring (European Parliament, 2006; Practice Committee of the American Society for Reproductive Medicine & the Practice Committee of the Society for Assisted Reproductive Technology, 2013). In more recent years, many authors have been discussing the expansion of hemoglobinopathies to all ethnic backgrounds given the presence of hemoglobin anomalies in multiple ethnic groups and the difficulties of clearly establishing the ethnic group of an individual. The 2021 ASRM/SART recommendations establish that hemoglobinopathies screening should be offered to all individuals, a practice that our center adhered to in 2021, after the period of the study presented in this manuscript. In further studies by our center, we would like to understand whether this modification in our practices results in a significant increase in diagnosing of the carrier state for hemoglobinopathies or whether no alterations in frequency are found.

In our study, it was offered standard genetic counseling and basic carrier screening, studying pathogenic variants for SMA and *CFTR*-related cystic fibrosis, karyotype, *FMR1* in females, and performing hemoglobin electrophoresis in some candidates. With this strategy, we disclosed relevant findings in 21.7% of candidates, and 16.5% had a relevant finding in their carrier screening, even if not all related with an inherited condition, such as our patient who was diagnosed with CML after the identification of a Philadelphia chromosome. Most studies related to genetic counseling in gamete donation focus on the findings of the genetic carrier screening, without describing the findings of the personal and familial history as reported here. With this report we hope to highlight how important this evaluation is, and how diverse the findings in this step can be.

As more authors are discussing the inclusion of a long list of genes for carrier screening, as an expanded carrier screening (ECS), it is expected that the proportion of candidates with findings would increase, with studies describing carrier rates of 24%-41%, listing different screened genes (Lazarin *et al*., 2013; Mertes *et al*., 2018; Payne *et al*., 2021; Urbina *et al*., 2017). A donor-recipient genetic matching system, to decrease the probability of combining two affected alleles in autosomal recessive disorders, has been proposed, and it is recommended in some countries, such as in Spain (Castilla *et al*., 2020). ECS also will predictably increase the workload of health professionals responsible for genetic counseling. Organizations planning on performing ECS should reflect before implantation of this strategy, whether their personal has the appropriate number and training to deal with this new paradigm.

Some authors believe that ECS should not be mandatory in the gamete donation process, given the high yield of findings but with low probability of having offspring with one of the screened conditions, and the negative psychological impact that this screening might bring to candidates (Pennings, 2020). In our study, no candidate withdrew from the donation process, pre-test or post-test, due to the psychological burden of the knowledge of being a carrier of a genetic disorder. Conversely, for some candidates with a positive family history of an autosomal dominant disorder, the pre-test genetic counseling was an opportunity to be voluntarily oriented for further care. Notwithstanding, in our pre-test consent form, candidates could opt not to know whether they had a specific alteration in their genetic screening, and before the findings being disclosed, they were inquired again about their willingness to know their carrier state. All candidates opted to know their findings.

In our work, we expanded the knowledge in the field of genetic counseling in gamete donation by reviewing whether family members were counseled after the identification of a pathogenic variant in an index case. In our center, a major difficulty for extending family counseling was the diverse geographical background of candidates, with family members often living abroad. For family members living in Portugal, extended counseling was possible through referral by their family doctor. Cascade counseling to parents, siblings, and partners allowed carrier screening in more individuals with an impact on the reproductive options of the family, beyond the index-case initially tested.

## CONCLUSIONS

Pre-test genetic counseling is a pivotal step in the gamete donation process and its standard operating procedure should be a topic of future discussion in the scientific community.

With a minimal genetic screening approach, 11/97 candidates showed to be carriers of autosomal recessive conditions. It is expected that if an ECS strategy is adopted more candidates will get a positive carrier screening, raising concerns with strategies to match with the recipient and increased workload. Strategies to implement ECS should be thought in advance.

Genetic counseling for family members is an important topic of discussion in the case of pre-test and post-test findings. Each center performing gamete donation should consider defining a plan of action for these situations.

## References

[r1] Amor DJ, Kerr A, Somanathan N, McEwen A, Tome M, Hodgson J, Lewis S (2018). Attitudes of sperm, egg and embryo donors and recipients towards
genetic information and screening of donors. Reprod Health.

[r2] Assembleia da República (2005). Lei Nº 12/2005 de 26 de Janeiro. Informação
genética pessoal e informação de saúde.

[r3] Castilla JA, Abellán F, Alamá P, Aura M, Bassas L, Clúa E, De la Fuente A, Guillén J, Manau D, Ruedak J, Ruizl M, Vendrellm X, on behalf of the Spanish Fertility Society, the Spanish Association
of Andrology, the Spanish Association of Medical Biopathology and
Laboratory Medicine, the Association for the Study of Reproductive
Biology, the Spanish Association of Human Genetics (2020). Genetic screening in gamete donation: Recommendations from SEF,
ASESA, AEBM-ML, ASEBIR and AEGH. Med Reprod Embriol Clin.

[r4] Dondorp W, De Wert G, Pennings G, Shenfield F, Devroey P, Tarlatzis B, Barri P, Diedrich K, Eichenlaub-Ritter U, Tüttelmann F, Provoost V (2014). ESHRE Task Force on Ethics and Law 21: genetic screening of
gamete donors: ethical issues. Hum Reprod.

[r5] European Parliament (2006). Commission Directive 2006/17/EC. ANNEX 3. Selection criteria and
laboratory tests required for donors of reproductive cells as referred to in
article 3(b) and article 4(2).

[r6] Jorge P, Oliveira B, Marques I, Santos R (2013). Development and validation of a multiplex-PCR assay for X-linked
intellectual disability. BMC Med Genet.

[r7] Lazarin GA, Haque IS, Nazareth S, Iori K, Patterson AS, Jacobson JL, Marshall JR, Seltzer WK, Patrizio P, Evans EA, Srinivasan BS (2013). An empirical estimate of carrier frequencies for 400+ causal
Mendelian variants: results from an ethnically diverse clinical sample of
23,453 individuals. Genet Med.

[r8] Mertes H, Lindheim SR, Pennings G (2018). Ethical quandaries around expanded carrier screening in
third-party reproduction. Fertil Steril.

[r9] Ministério da Saúde (2014). Decreto-Lei N.º 131/2014. Regulamenta a Lei N.º 12/2005.

[r10] Payne MR, Skytte AB, Harper JC (2021). The use of expanded carrier screening of gamete
donors. Hum Reprod.

[r11] Pennings G (2020). Expanded carrier screening should not be mandatory for gamete
donors. Hum Reprod.

[r12] Practice Committee of the American Society for Reproductive Medicine
and the Practice Committee of the Society for Assisted Reproductive
Technology (2013). Recommendations for gamete and embryo donation: a committee
opinion. Fertil Steril.

[r13] Practice Committee of the American Society for Reproductive Medicine
and the Practice Committee for the Society for Assisted Reproductive
Technology (2021). Electronic address: ASRM@asrm.org. Guidance regarding gamete and
embryo donation. Fertil Steril.

[r14] Urbina MT, Benjamin I, Medina R, Jiménez J, Trías L, Lerner J (2017). Expanded carrier screening in gamete donors of
Venezuela. JBRA Assist Reprod.

